# Lymphocytic Enterocolitis in Systemic Lupus Erythematosus

**DOI:** 10.4103/1319-3767.56100

**Published:** 2009-10

**Authors:** Mohamed O. Hegazi, Salem F. Owayed, Mohamed Mourou, Munish Joneja, Anant Mashankar

**Affiliations:** Department of Medicine, Al Adan Hospital, Kuwait; 1 Yaco-Al Adan Radiology and Laboratory Center, Kuwait

**Keywords:** Lymphocytic, microscopic colitis, systemic lupus erythematosus

## Abstract

Microscopic colitis (MC) is a recognized cause of chronic watery diarrhea. It is characterized by subepithelial collagen deposition or intraepithelial lymphocytic infiltration of the colonic mucosa which, however, appears grossly normal on endoscopy. The term microscopic enterocolitis is applied when MC is associated with similar microscopic affection of the ileum and/or proximal small intestine. MC is reported to be associated with a variety of autoimmune conditions. Systemic lupus erythematosus (SLE) is rarely reported in association with MC. We report a female patient with microscopic enterocolitis as one of the presenting manifestations of SLE.

Gastrointestinal (GI) manifestations of systemic lupus erythematosus (SLE) are common; however, they are frequently overlooked in the presence of renal, pulmonary, and cerebral complications.[1] These manifestations include mouth ulcers, dysphagia, anorexia, nausea, vomiting, hemorrhage, and abdominal pain.[1] Protein-losing enteropathy in patients with SLE has also been mentioned in a small number of reports and, in most of these cases, endoscopy and mucosal biopsy were reported to be normal.[2,3] Microscopic colitis (MC) is a recognized cause of diarrhea with a normal endoscopic appearance of the colonic mucosa.[4] MC is reported to be associated with a variety of autoimmune diseases, however its occurrence with SLE is relatively uncommon.[5] We report a female patient who had chronic diarrhea due to microscopic enterocolitis as a presenting feature of SLE.

## CASE REPORT

A 54-year-old lady presented with diarrhea of 3 weeks' duration. She gave history of intermittent watery diarrhea over the last 2 years. The diarrhea was nonbloody, without nocturnal symptoms, and associated with significant weight loss. Two years earlier, she had been diagnosed as a case of primary antiphospholipid syndrome after she developed a deep vein thrombosis in her left leg and she had been advised to take life-long warfarin therapy.

**Figure 1:**
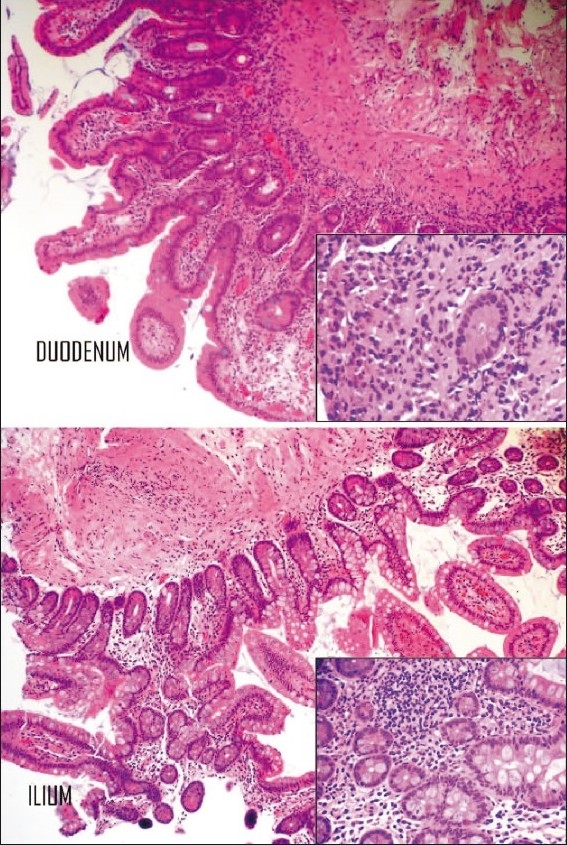
Low- and high-power (inset) fields of duodenal and ileal biopsies showing intraepithelial and subepithelial lymphocytic infiltrates

Clinically, she was pale and underweight. Examination of the chest showed a left pleural effusion. Apart from sparse scalp hair, she did not have any other clinical stigmata of autoimmune or collagen disease. Pleural aspirate showed an exudative effusion with positive antinuclear antibody in the pleural fluid. Stool microscopy and culture were negative. Blood investigations showed: hemoglobin: 9.6 gm/dl, mean corpuscular volume: 81 fl, total leukocyte count: 4.5 × 10^9^/l, platelets: 130 × 10^9^/l, 24-h urinary protein: 450 mg, serum albumin: 32 gm/l, serum folate: 5.2 ng/ml (normal >2 ng/ml), serum iron: 72 *μ*g/dl (normal: 65–176 *μ*g/dl), serum ferritin: 43 ng/ml (28–201 ng/ml), and serum B_12_: 378 pg/ml (normal 200–900 pg/ml). Hepatic and renal function tests were normal. Anticardiolipin, antinuclear, anti-DNA, anti-Ro, anti-RNP, and anti-Sm antibodies were positive. Celiac disease serology, including antiendomysial, antigliadin, and tissue transglutaminase antibodies, was negative both initially and also when repeated 6 months later for confirmation. Both esophagogastroduodenoscopy and colonoscopy showed normal mucosal appearance. Small bowel biopsy showed mild blunting of the villi, with epithelial and subepithelial lymphocytic infiltration [Figure 1]. Biopsies from various parts of the colon and from the terminal ileum showed intraepithelial and subepithelial lymphocytic infiltration suggestive of lymphocytic colitis [Figure 1]. With the presence of only mild villous blunting in the duodenal biopsy (and not total villous atrophy), negative celiac disease serology (which is positive in more than 95% of cases of celiac disease), normal values of serum iron and folate,_,_ and the typical endoscopic and histopathologic criteria of MC, we arrived at the diagnosis of lymphocytic enterocolitis. A collective diagnosis of SLE, antiphospholipid syndrome, and microscopic ‘lymphocytic’ enterocolitis was made. Considering the coexistence of MC with other manifestations of SLE, oral prednisolone 1 mg/kg/day was started; loperamide was prescribed for symptomatic relief.

The patient's diarrhea improved with oral prednisolone. Later, the dose of prednisolone was gradually tapered to a maintenance dose of 15 mg/day and hydroxychloroquine 200 mg twice daily was added as part of the therapy for SLE. She remained free of diarrhea while on the smaller maintenance dose of steroids.

## DISCUSSION

MC is characterized by chronic watery (secretory) diarrhea without bleeding.[4] Although the colon appears normal on colonoscopy, there is histopathological evidence of inflammation.[4,5] Data regarding the epidemiology of MC are limited. In one of the largest population-based studies that was conducted in Barcelona, MC was more common in females and was diagnosed at a rate of 9.5 per 100 apparently normal colonoscopies performed in patients with watery diarrhea.[6] Theories on pathogenesis suggest that abnormal collagen metabolism, use of nonsteroidal anti-inflammatory drugs (NSAIDs), bacterial toxins, or diabetes mellitus may be possible etiologic factors.[4,5] The clinical course may be chronic intermittent (80%) or chronic continuous (12%), or there may be only a single episode (8 %).[4] The diagnosis is established by biopsy of the colonic mucosa, which reveals colitis but not mucosal ulcerations.[4,5] MC is divided into two subsets according to histopathological appearance: collagenous colitis, with colonic subepithelial collagen deposition, and lymphocytic colitis, with intraepithelial lymphocytic infiltrates but no collagen deposition.[4,5] In addition to the colonic affection, other parts of the GI tract, especially the ileum, are reported to be similarly affected.[7,8] Sapp *et al*. studied terminal ileal involvement and concluded that intraepithelial lymphocytosis occurs in the terminal ileum in patients with MC.[7] DuBois *et al*, described three patients with lymphocytic colitis and celiac disease–like changes of the small bowel who failed to respond to a gluten-free diet, and suggested that involvement of the small bowel in lymphocytic colitis may represent a distinct panintestinal disease for which the term ‘lymphocytic enterocolitis’ was proposed.[8] Our patient had lymphocytic enterocolitis with affection of the colon and terminal ileum as well as of the proximal small bowel. An association between MC and celiac disease has been observed.[4,5] MC has also been reported in association with a variety of autoimmune diseases, including autoimmune thyroid disease, pernicious anemia, autoimmune hemolytic anemia, ankylosing spondylitis, rheumatoid arthritis, Sjögren syndrome and, rarely, SLE.[4,5] In a review by Chande *et al*., among 104 patients with MC, 30 (29%) had at least one associated autoimmune disease; SLE was found in only one case.[5] In addition to presenting with a rarely reported association, our patient had lymphocytic enterocolitis as one of the presenting features of SLE. Treatment options for MC include cholestyramine, sulphasalazine, and oral budesonide or prednisolone, in addition to stopping the offending drug (e.g., NSAID).[9,10] Surgery (ileostomy, sigmoidostomy, or colectomy) has been undertaken occasionally for the treatment of microscopic colitis.[10]

In conclusion, MC should be considered in the differential diagnoses of diarrhea in patients with SLE. The endoscopic appearance is normal in MC, and colonic mucosal biopsy is required to establish the diagnosis. In cases of MC, similar microscopic involvement of the small bowel makes the term microscopic enterocolitis more appropriate. The symptoms of MC may respond to steroids given to treat the associated autoimmune disease.
